# College and Hospital Notes

**Published:** 1906-11

**Authors:** 


### COLLEGE AND HOSPITAL NOTES.

The United States Military Hospital, at the Presidio, San Francisco, was destroyed by fire on the evening of October 13, 1906. The building was crowded with patients at the time, some of whom were critically ill, but all were removed in safety, not a single casualty being reported. The fire was due to the explosion of a gasoline tank in the rear of the building, from which the flames spread with alarming rapidity. The men of the Twentieth Infantry and four troops of the Fourth Cavalry stationed at the Presidio responded speedily to the call and through their efforts the fire was limited to the building where it started. The structure was comparatively new and cost about $50,000. The loss will be especially felt at this time, hospital accommodations in San Francisco being limited, because of the earthquake last spring.

